# An Ultra-Low Power Surface EMG Sensor for Wearable Biometric and Medical Applications

**DOI:** 10.3390/bios11110411

**Published:** 2021-10-21

**Authors:** Yi-Da Wu, Shanq-Jang Ruan, Yu-Hao Lee

**Affiliations:** 1 Department of Electronic and Computer Engineering, National Taiwan University of Science and Technology, Taipei 106, Taiwan; m10802114@mail.ntust.edu.tw (Y.-D.W.); sjruan@mail.ntust.edu.tw (S.-J.R.); 2 Department of Physical Medicine and Rehabilitation, Shuang Ho Hospital, Taipei Medical University, Taipei 106, Taiwan

**Keywords:** EMG acquisition system, biosensor devices and interface circuit, wireless transmission, power consumption

## Abstract

In recent years, the surface electromyography (EMG) signal has received a lot of attention. EMG signals are used to analyze muscle activity or to evaluate a patient’s muscle status. However, commercial surface EMG systems are expensive and have high power consumption. Therefore, the purpose of this paper is to implement a surface EMG acquisition system that supports high sampling and ultra-low power consumption measurement. This work analyzes and optimizes each part of the EMG acquisition circuit and combines an MCU with BLE. Regarding the MCU power saving method, the system uses two different frequency MCU clock sources and we proposed a ping-pong buffer as the memory architecture to achieve the best power saving effect. The measured surface EMG signal samples can be forwarded immediately to the host for further processing and additional application. The results show that the average current of the proposed architecture can be reduced by 92.72% compared with commercial devices, and the battery life is 9.057 times longer. In addition, the correlation coefficients were up to 99.5%, which represents a high relative agreement between the commercial and the proposed system.

## 1. Introduction

An increasing amount of research on combining bioelectrical signals with the Internet of Things (IoT) has been undertaken in the last decade. More biometric sensor products have been developed, with the most popular product being the surface electromyogram (EMG). Surface EMGs have been used extensively in various applications such as games, rehabilitation medicine, analysis of motion, analysis of muscle fatigue, and prosthesis control [1,2,3,4]. The surface EMG sensor is able to record muscle activity by using EMG electrodes to measure the changes in the electrical potential between two points of a muscle [5]. Furthermore, the sensor drastically decreases bacterial infection risk because of its non-invasive measurement [6].

Comparisons of commercial products have found that although many surface EMG sensors on the market claim to be low-power and high-sampling, the lowest power consumption among these products is up to 46.25 mW [7,8,9,10]. The battery life depends on the battery capacity, and the maximum battery life is only 8 h. Therefore, many researchers have investigated low-power and high sampling surface EMG systems for long-term recording and applied them in different fields. Brunelli et al. [4] developed a wireless multi-channel surface EMG prosthetic sampling measurement system, using 240 Kbps speed Bluetooth technology. The research used 32-channel surface EMG sensors to sample gesture signals. The gesture signals are extracted and classified as features of hand movement and surface EMG signals are used to control the complex gestures of prosthetic hands. However, the power consumption was found to be as high as 160 mW. Giorgi et al. [11] implemented a wireless surface EMG and accelerometer signal sensing system. The system combines surface EMG sensors with accelerometers and is used for muscle fatigue detection. The results of the research show that when athletes wear this system device, they obtain better training results by correcting the muscle angle to obtain more strength. The device uses ZigBee wireless transmission technology and transmits data at a speed of 2 Ksps, but the power is as high as 169.3 mW. The two examples above demonstrate that the surface EMG sampling system has been successfully implemented. They have been optimized for use in different fields, and have greater application for daily use than commercially available products. However, they still suffer from the problem of high-power consumption.

**Figure 1:**
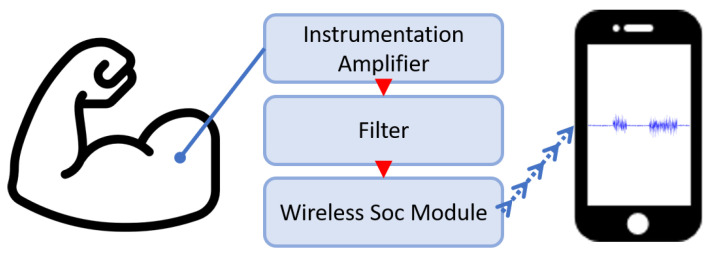
Overview of the proposed surface EMG acquisition system.

The purpose of this study was to implement an ultra-low-power surface EMG acquisition system for high-sampling measurement. In the circuit, we designed a wireless surface EMG acquisition system that consists of an instrumentation amplifier, an analog filter, and a wireless Soc module. In regard to the analog filter, we studied and optimized the common filter architecture. We used a Bluetooth low energy (BLE) module as the wireless SoC module, and embedded a ping-pong buffer mechanism on the memory architecture. The ping-pong buffer is a power-saving mechanism; it also improves the BLE data transmission efficiency. The data is transmitted by the BLE module to the smartphone. The smartphone uses an infinite impulse response filter (IIR filter) to remove noise and display the data on the screen (Figure 1).

The remainder of this paper is organized as follows. Section 2 describes the details and functionality of various components used in the sensor. We design the instrumentation amplifier and the analog filter for the surface EMG signal processor analog circuit and we propose a power-saving method based on a ping-pong buffer as the memory architecture in Section 3. The experimental results are presented and discussed in Section 4. Finally, the conclusion is provided in Section 5.

## 2. Surface EMG Architecture

**Figure 2:**
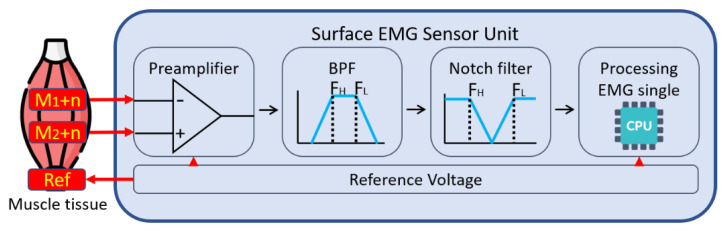
Surface EMG measurement sensor block diagram. The EMG signal is represented by ‘M’ and the noise signals by ‘n’.

Surface EMG measurement usually uses a preamplifier to output the voltage difference between the muscle voltage at two points to the next stage. When the EMG signal passes through various tissues, various noises will interfere with the signal. Therefore, a band-pass filter (BPF) and notch filter are used to remove this noise. Lastly, a microcontroller unit (MCU) with an analog-to-digital converter (ADC) is used to collect the surface EMG signals (Figure 2).

### 2.1. Instrumentation Amplifier

The preamplifier is a key element in the bioelectric signal measurement system, and an instrumentation amplifier (In-Amp) is usually used as the preamplifier. The In-Amp is improved by a differential amplifier so that it has low DC offset ($V_{os}$) and low noise. In the surface EMG measurement system, a common-mode rejection ratio (CMRR) of more than 100 dB, a high bandwidth (BW), and an input impedance ($Z_{in}$) greater than 100 GΩ are required, at least [12,13,14].

### 2.2. Processing Surface EMG Noise of Filter

#### 2.2.1. Active Filter

The active filter has good isolation between the two stages. It can provide high input impedance and low output impedance, and it is convenient for connecting with the front and rear circuits. Compared with passive filters, the active filter does not have to take into account each stage’s frequency-dependent loading of the preceding stage. Active filters can adjust the sensitivity of the frequency response by simply setting the Quality Factor (Q Factor). Usually, the Q factor is designed to be 0.707 because the signal output will have the best flattening effect [15,16,17].

**Figure 3:**
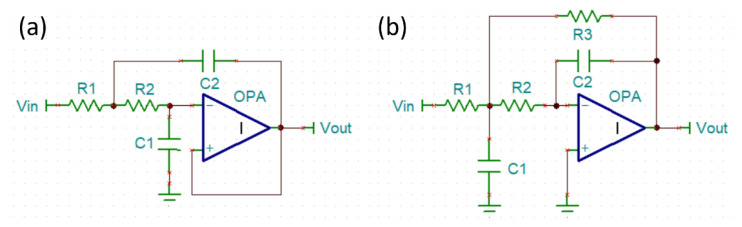
(**a**) Sallen–Key filter architecture (**b**) MFB filter architecture.

**Figure 4:**
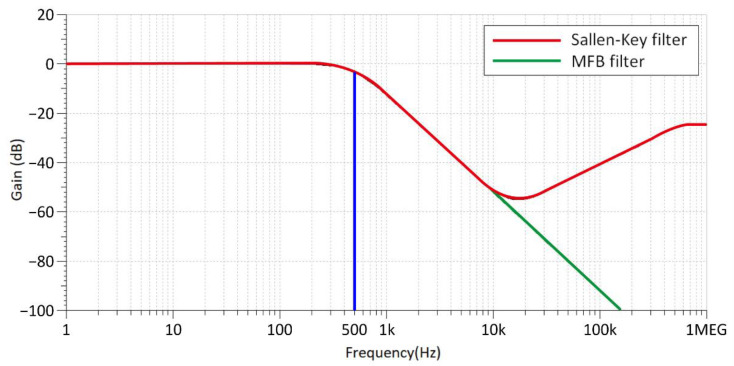
Frequency response of the MFB filter architecture and Sallen–Key filter.

Many documents or articles suggest using a Sallen–Key filter to design surface EMG sensors [18,19,20]. The Sallen–Key filter has simple architecture, an uncomplicated design, and can be constructed with a few electronic components (Figure 3a). However, high-frequency signals that are higher than the cut-off frequency (Fc) can easily pass through the feedback capacitor (C2) and directly output to the next stage. High-frequency signals that cannot be eliminated will increase unnecessary power consumption. Therefore, we proposed the use of the multiple feedback filter (MFB filter) to effectively solve the problem of high frequency signal transmission to the next stage (Figure 3b). Then, we experimented with the frequency response of the Sallen–Key filter and MFB filter in low-pass mode (Fc = 500 Hz). Figure 4 shows the confirmed and high frequency signals beyond Fc of the Sallen–Key filter, which cannot be filtered. On the other hand, the MFB filter is able to filter out the high frequency signal perfectly.

#### 2.2.2. Notch Filter

The range of the EMG effective frequency is low (about 20 Hz~500 Hz) [21,22], so the noise will easily affect the EMG signal. Further, the surface EMG signal will generate a lot of noise when passing through different muscle tissues; these noises can be divided into the following types:

- Power line noise

The most serious interference in surface EMG measurement is electromagnetic interference, which is caused by AC power lines. Its main frequency component is 60 Hz [23] because power line noise is within the effective range of surface EMG. Some scholars advocate using a notch filter to filter this interference [24,25]. However, other scholars have different opinions because 30 Hz~300 Hz is the densest frequency band of EMG signals [26]; thus, if a notch filter works, important signals may also be filtered out. Therefore, they do not advocate such an approach [18,27,28]. As a result, IIR and power spectral density (PSD) compensation have been designed to separate power line noise and surface EMG signals more effectively.

- Inherent instability of signal

The EMG signal can be affected by the motor units. In most conditions, these frequency regions are 0 to 20 Hz. In this case, the noise is considered as unnecessary, so it is essential to remove it cleanly.

### 2.3. Storage Method of Wireless Embedded System

#### 2.3.1. The Use of Wireless Transmission Technology

Surface EMG signal detection can be achieved by utilizing wireless technologies. Many wireless transmission technologies are available today, such as 2G/3G wireless networks, LoRa, Zigbee, etc. However, 2G/3G wireless networks are costly and consume high amounts of power, and LoRa and Zigbee have low data rates. Therefore, BLE and Wi-Fi are the most popular wireless technology used in our daily life. In this paper, Wi-Fi modules and BLE modules with SoC are investigated and evaluated based on their data throughput. Wi-Fi technology, for example, ESP8266EX [29] has a data transmission speed of 4.5 MB/s and an average current of 80 mA, while the power consumption is much lower for BLE technology than Wi-Fi. As a result, we used nRF52832 [30] as a wireless transmission platform. When the DC–DC low power consumption mode is enabled, the average current is only 1.32 mA and the data transmission speed can reach 171.5 KB/s.

#### 2.3.2. Improvement in Data Processing Efficiency Based on Ping-Pong Buffer

**Figure 5:**
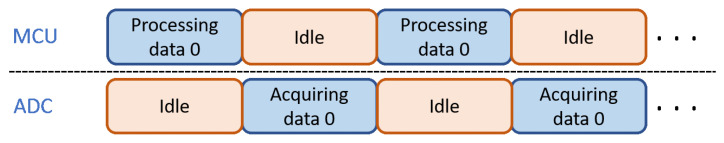
The original time graph with the FIFO.

**Figure 6:**
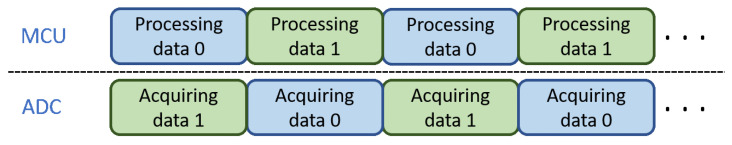
Improved time graph with the ping-pong buffer.

Figure 5 describes the common ADC hardware design for the first input first output (FIFO) process of data allocation. After the MCU finishes processing the surface EMG signal data, the MCU calls on the ADC hardware to start collecting data, and MCU waits for the Interrupt Service Routine (ISR) to be triggered. When the ADC hardware finishes acquiring data, the MCU processes the ADC data, and the ADC hardware waits for the MCU to finish processing data again, repeating this action indefinitely. However, this kind of situation will lead to discontinuous collection of signals, lower system utilization and wasted power consumption. In order to make sure data will not be lost, the surface EMG measurement system needs high stability and timely transmission. Hence, we embedded a ping-pong buffer mechanism [31,32,33,34] in the MCU architecture (Figure 6).

## 3. Implementation Method

**Figure 7:**
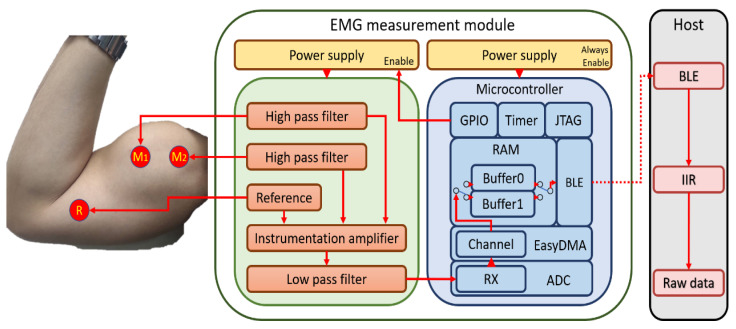
Diagram of the proposed surface EMG measurement module system.

In this paper, we propose a surface EMG measurement module and a smartphone as a host (Figure 7). The surface EMG measurement module captures the analog EMG signal on the skin, which is sent via the biceps brachii muscles to the surface electrodes. Then, the EMG data are transmitted to the host using wireless for further processing.

### 3.1. Measurement Module Design

#### 3.1.1. Instrumentation Amplifier with Passive High-Pass Filter

We searched the literature to compare the commonly used low-power In-Amps, and chose the INA333 device [35,36] as our In-Amp. The INA333 is much better than the AD620 [37,38] and INA128 [39,40] in $Z_{in}$. Although the quiescent current ($I_{Q}$) and $Z_{in}$ of the INA333 are 10 μA and 10 GΩ higher than that of the AD8236 [41,42,43], the IN333 has better performance with regard to BW, CMRR, noise and $V_{os}$ (Table 1). 

**Figure 8:**
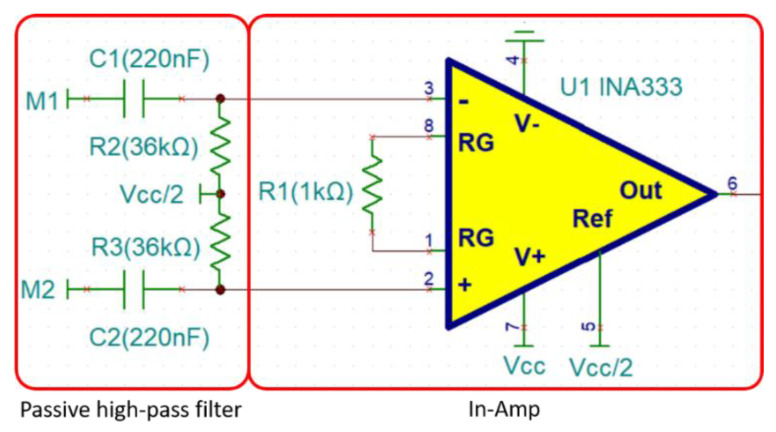
In-Amp with passive high-pass filter architecture.

The INA333 requires a high resistor (*R*_2_ and *R*_3_) on the input pin to form the input bias current return path (Figure 8). This approach results in a better high-frequency CMRR and lower $V_{os}$ [35,44,45]. In regard to the In-Amp power supply, the In-Amp is designed for single supply mode. Since the EMG signal has both positive and negative signals, the input bias current return path provides a bias voltage (Vcc/2) and allows the EMG signal to float on the bias voltage [44,46]. We increased the capacitors in the input path and formed a first-order passive high-pass filter by adding resistance. The first-order passive high-pass filter’s Fc is designed to 20 Hz. The equation for the first-order passive high-pass filter is shown in Equation (1):

(1) $$\mathrm{Fc}=\frac{1}{2\pi RC}.$$

**Figure 9:**
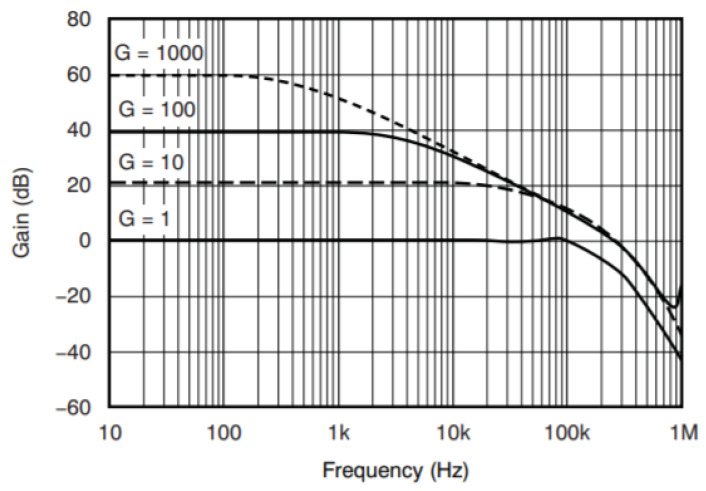
Gain vs. frequency.

The In-Amp’s gain is determined by the value of *R*1. Since the maximum frequency of EMG is 500 Hz, and according to Nyquist sampling theorem, the stable signal needs to be at 1 KHz. When the gain of INA333 is at 100 (Figure 9), signals within 1 KHz can be kept stable as shown in the Equation (2):

(2) Gain=1+(100 KΩ/R1).

#### 3.1.2. Design of the MFB Low-Pass Filter

**Figure 10:**
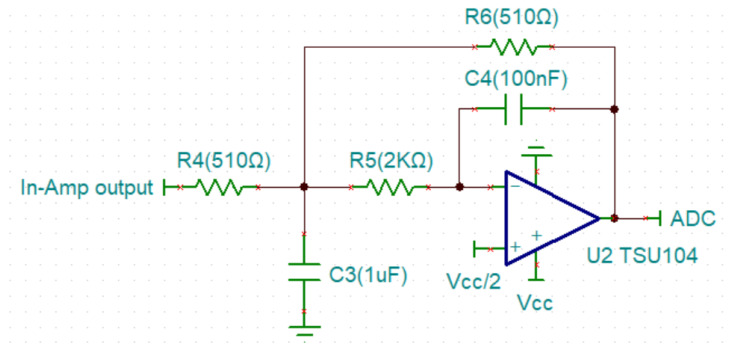
Architecture of the MFB low-pass filter.

The appropriate bandwidth of an EMG signal is maximized to 500 Hz, as mentioned in Section 2.2.2. We chose a 1 μF capacitor for $C_{3}$ and 0.1 μF capacitor for $C_{4}$ in a low-pass filter, then the Okawa Electric Design calculator was utilized to compute the parameters and the results for $R_{4}$ and $R_{6}$ were 506.605 Ω and $R_{5}$ was 2 kΩ (Figure 10). Because the closest to 506.6 Ω on the market was 510 Ω, we chose 510 Ω to replace 506.6 Ω. After re-calculating, we designed the actual Fc of the filter to be 498.33 Hz. We used the error percentage of our desired values and compared them to our actual values. Finally, we found that our Fc had an error of 0.334% as calculated by Equation (3):

(3) $$\delta =\frac{\left|a-b\right|}{\left|a\right|}\cdot 100\%,$$

where δ is the percent error, a is the theoretical value and b is the experimental value. In this paper, the error rate of resistance and capacitance is 1%. Therefore, an error percentage below 1% is reasonable. According to Section 2.2.1, the Q factor of 0.707 is calculated by Equation (4):

(4) $$Q\;\mathrm{factor}=\frac{\sqrt{\frac{C_{3}}{C_{4}}}}{\sqrt{\frac{R_{6}}{R_{5}}}+\sqrt{\frac{R_{5}}{R_{6}}}+\sqrt{\frac{R_{5}R_{6}}{R_{4}}}}.$$

The result of the Q factor is 0.708 (the error percentage = 0.141%).

The filter outputs an unstable signal in the initial state because it takes some time before the capacitor is stable. To calculate the instability time, we used the Laplace transfer to analyze the transient response. The Laplace transfer function of the MFB low-pass filter is shown in Equation (5):

(5) $$\frac{V_{o}}{V_{i}}=\frac{-1}{C_{3}C_{4}R_{4}R_{5}}\cdot \frac{1}{s^{2}+s\frac{1}{C_{3}R_{4}R_{5}}\left(R_{4}+R_{5}\left(1+\frac{R_{4}}{R_{6}}\right)\right)+\frac{1}{R_{5}R_{6}C_{3}C_{4}}}.$$

**Figure 11:**
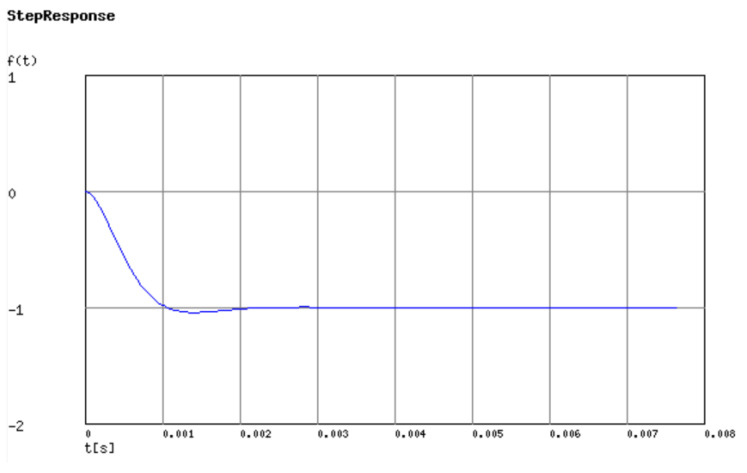
Transient analysis of the proposed MFB filter.

Figure 11 shows that the capacitor is stable after 2 milliseconds. In order to ensure a clean signal, the surface EMG signal in our work is collected after 10 milliseconds.

#### 3.1.3. Central Control Unit

##### Saving-Power Mechanism

The MCU clock rate and the square of the MCU voltage are proportional to the dynamic power, and what they consumed is as shown in Equation (6):

(6) $$P_{d}=CV^{2}f,$$

where $P_{d}$ is the dynamic power, C is the switched load capacitance, V is the supply voltage, and f is the MCU adjustable clock rate. To simultaneously reduce the power consumption and finish data transmission, it is essential to minimize the MCU frequency. Hence, it is proposed to use two different frequency crystal oscillator sources in the MCU—the 64 MHz high-frequency clock (HFCLK) used in BLE transmission, and the 16 MHz low-frequency clock (LFCLK) is used by the hardware ADC in the acquisition. Besides, the LFCLK is enabled when the ADC signal is collected to keep the power consumption at a minimum. When the EMG signals fill up the buffer, HFCLK will be activated, and the data will be sent to the host by BLE. This design method can effectively save power. In addition, we used two low dropout regulators (LDO) in our system, one is always enabled for the MCU power supply, and the other is controlled by the MCU. When the host starts to get data, the GPIO of the MCU becomes high to enable the LED and start to collect EMG signals. Conversely, when the host command does not need to obtain data and the GPIO is at a low level, the disabled LDO forcibly stops collecting EMG signals, and the MCU enters the idle mode. After the MCU enters the idle mode, the overall average current will remain at 1.9 uA.

##### Storing Surface EMG Data by the Ping-Pong Buffer Mechanism

**Figure 12:**
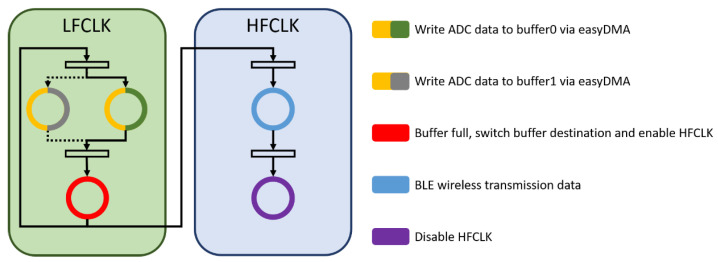
The proposed two MCU clock sources with ping-pong buffer architecture.

According to the Nyquist sampling theorem, the minimum sampling frequency is 1 kHz. In this paper, the MCU was designed to acquire a single channel of 2.049 kHz, the sampling resolution was 14 bits, and the easy-to-use direct memory access (EasyDMA) interrupt trigger was used. In order to avoid data loss, the digital EMG data will be pushed into one of the ping-pong buffers instead of being directly transmitted to the host via BLE. When the buffer is full, an interrupt will be triggered, and the buffer address will also be switched to other buffers. The full buffer transmits data to the host by going through the BLE (Figure 12).

### 3.2. The Host Processing with IIR

To ensure the data frequency is as concentrated as possible in the EMG signal range, we used two IIR filters to process the EMG signals. One is an eight-order digital Butterworth IIR band-pass filter that is used to eliminate noise, and the other is a fourth-order digital Butterworth IIR band-stop filter that is used to eliminate the main frequency component of the power line at 60 Hz. The passband range and the stopband range are 20 Hz to 500 Hz and 55 Hz to 65 Hz, respectively. The equation for the IIR filter is shown in Equation (7):

(7) $$y\left[n\right]=\overset{K}{\underset{K=0}{\sum }}b_{k}x\left[n-k\right]-\overset{L}{\underset{l=1}{\sum }}a_{l}y\left[n-l\right],$$

where y[n] is the output signal, K is the feedforward filter order, $b_{k}$ is the feedforward filter coefficient, x[n] is the input signal, L is the feedback filter order, and $a_{l}$ is the feedback filter coefficient. The utilization of FIR-based filters can avoid arithmetic divisions by using the least-square strategy, but they need a higher order to get the same attenuation slope as the IIR filter. Hence, the IIR filter was chosen as the noise filtration method for its lower power consumption [47,48].

## 4. Results

**Figure 13:**
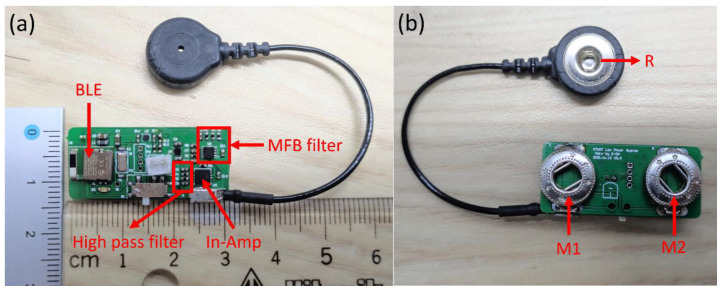
(**a**) Front side and (**b**) back side of the ultra-low power surface EMG signal acquisition system.

In this paper, the ultra-low power surface EMG signal acquisition system was designed as shown in Figure 13. To verify the reliability of the proposed system, the signal-to-noise ratio (SNR), linear correlation and power consumption were calculated, and a commercial wireless EMG detection system was used as the criterion reference [7].

### 4.1. SNR

SNR is the ratio of signal power to the noise power, and it is a form of measurement used in certain applied sciences. If the SNR ratio is high, it indicates that a better signal quality is obtained. For example, a high SNR ratio in audio systems means better sound quality. Therefore, we used it as a measure of the quality of the EMG signal. To calculate SNR, we defined 20 to 500 Hz as the desired signal, other signals as noise, and input 10 mVp-p Gaussian white noise to the device channel to simulate the noise immunity of the sensor. Previous studies reveal that an adequate SNR should be at least 18 dB in a surface EMG sensor [49,50]. The calculation for the SNR of our proposed system was 23.1 dB. This proves that our proposed system not only reduces costs, but also achieves adequate noise immunity.

### 4.2. Linear Correlation Coefficient

**Figure 14:**
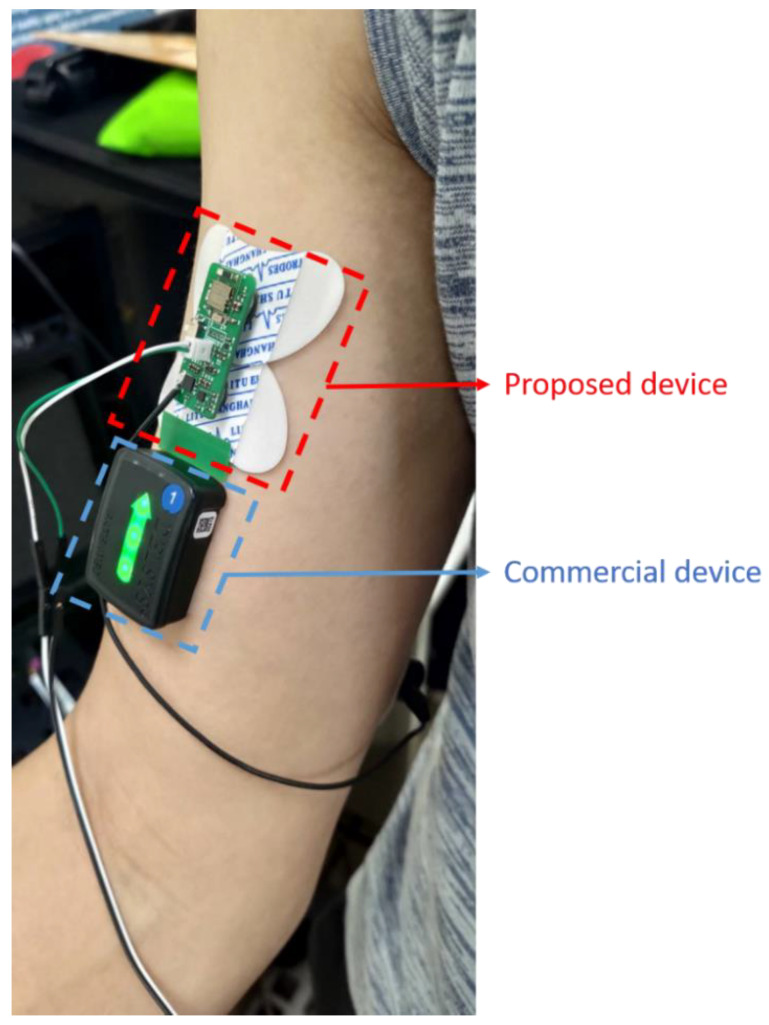
The electrode placement of the two systems.

**Figure 15:**
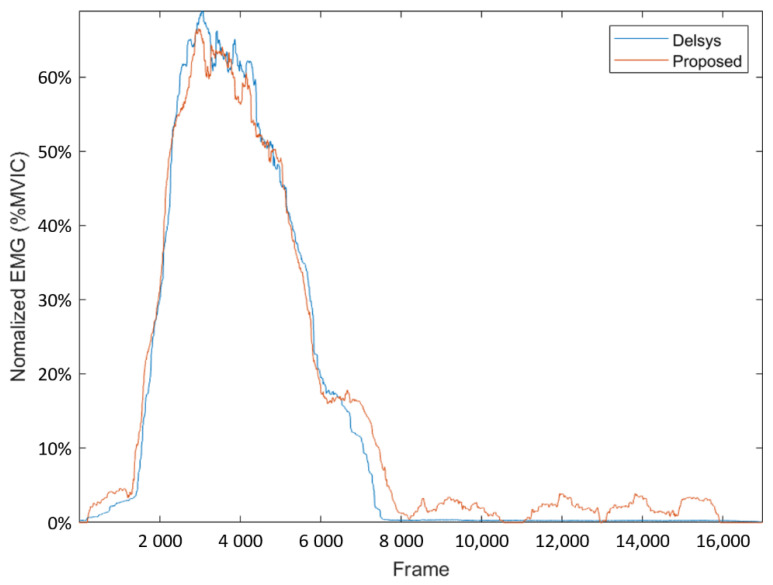
EMG signals from the commercial (blue) and the proposed device (orange).

In this part, we used the EMG signal of the measured biceps as a comparison. First, the mean absolute value (MAV) was obtained. The MAV ensures that the commercial device signals and proposed device signal scales are consistent. Then, the EMG signals from the proposed system and the commercial system were measured and processed using the same procedure, and compared to obtain the relative consistency between the two systems [51] (Figure 14). Finally, a comparison of the EMG signal for each system using the Pearson product-moment correlation coefficient (PPMCC) was used to illustrate the correlation of the waveforms. The correlations between the proposed system and the commercial system were as high as 0.995 (Figure 15).

### 4.3. Power Consumption

**Figure 16:**
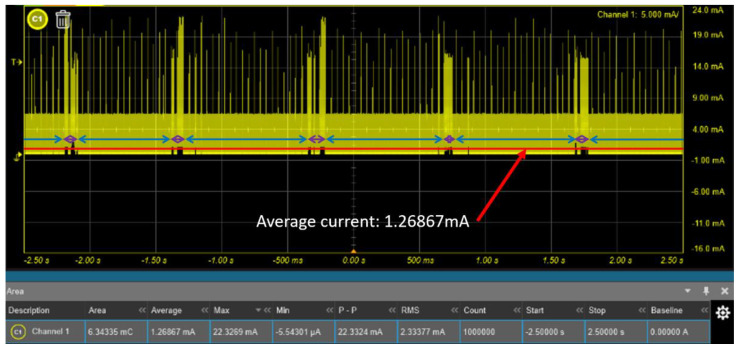
Current consumption of the surface EMG system. The blue line is only ADC conversion and the purple line is BLE transmission and ADC conversion.

**Figure 17:**
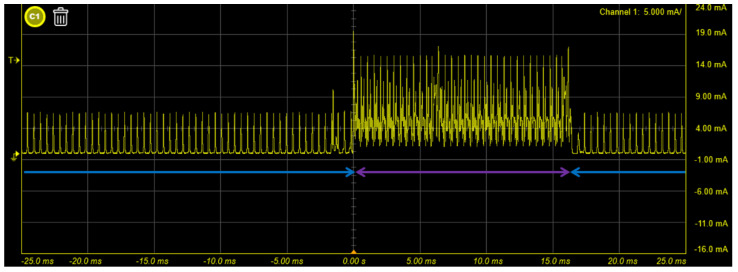
Current consumption of the surface EMG system for small time scales. The blue line is only ADC conversion and the purple line is BLE transmission and ADC conversion.

In this part, the current of the sensor was measured in three different states. First, when the sensor is not connected to the host and the EMG signal is not collected, the BLE will be in the advertising mode. In the advertising mode, BLE continuously sends advertisement packets through the 2402 MHz, 2426 MHz and 2480 MHz channels, and the host can quickly find the sensor and the connection. Consequently, the current is going to be higher; it will be approximately 5.3 mA. Second, after the host is connected to the sensor, it will fix the channels and change to the scan mode. Since the host has not yet started to collect EMG signals at this moment, the current is about 60.64 uA. Finally, when the host asks the sensor to collect EMG signals, the analog circuit and ADC will start operation, the current will increase to about 1.269 mA (Figure 16). Figure 17 depicts the reduced time scale and clearly shows the current change. We also measured the average current of the surface EMG sensor without the MCU, the average current is about 72.14 uA. Compared to the literature on surface EMG sensors without wireless communication, we achieved lower power consumption in our surface EMG sensors [52,53].

### 4.4. Advantages

Table 2 lists the key techniques used in the proposed design compared toother architectures. Compared with the previous works [54], the power consumption of our proposed architecture can be reduced by 92.46%. In addition, the battery life is 5.66 times longer than the previous work with the same 300 mAh lithium battery in a continuous wireless connection. Compared with commercial devices [7], our proposed system reduces the power consumption by 92.72% and the battery life is 9.057 times longer than the commercial devices [7].

## 5. Conclusions

In this paper, an ultra-low power surface EMG signal acquisition system was developed and we focused on optimizing each part of the surface EMG signal acquisition system. We confirmed that the power consumption and stability of the MFB filter was much better than the Sallen-Key filter suggested in the literature. Regarding the MCU power saving method, the system uses two different frequency MCU clock sources to satisfy these system requirements. In addition, we proposed a minimization method based on using a ping-pong buffer as the memory architecture to achieve the best power saving effect. Finally, the EMG signal was preserved by more effective noise removal through IIR. The experimental results showed that the proposed surface EMG sensor can significantly reduce power consumption. Compared with commercial devices, our proposed system reduced the power consumption by 92.72%. In addition, the collected EMG signals had a 99.5% high correlation with the commercial systems. Our proposed system was proven to be easily available and effective; it could be used for further research on other surface EMG applications, such as EMG posture recognition and control of a robotic arm.

## Figures and Tables

**Table 1:** Comparison of In-Amp parameters (gain = 100).

Chip	BW (kHz)	CMRR (dB)	$Z_{in}$ (GΩ)	Noise $\left(nV/\sqrt{\mathrm{Hz}}\right)$	$V_{os}$ (μV)	$I_{Q}$ (μA)
INA333	3.5	115	100	50	1	50
AD8236	0.8	110	110	76	2.5	40
AD620	120	130	10	28	50	900
INA128	200	120	10	8	50	700

**Table 2:** Comparison of various surface EMG hardware architectures.

Architecture	This Work	[54]	[7]
Wireless Technology	BLE	BLE	BLE
PCB Size (${\mathrm{cm}}^{2}$)	1.4 × 3.1	3.8 × 4.45	2.7 × 3.7
Battery (mAH)	300	300	N/A
Total Power Consumption	4.735 mW	62.7 mW	65 mW
Sampling Rate (Hz)	2k	1k	2.148k
SNR (dB)	23.1	N/A	65
Battery Life (hours)	63.4	11.2	7

## Data Availability

Not applicable.
